# The relationship between hypertension and sleep duration: an analysis of the fifth Korea National Health and Nutrition Examination Survey (KNHANES V-3)

**DOI:** 10.1186/s40885-015-0020-y

**Published:** 2015-06-11

**Authors:** Hye-Rim Hwang, Jeong-Gyu Lee, Sangyeop Lee, Kwang Soo Cha, Jung Hyun Choi, Dong-Wook Jeong, Yu-Hyun Yi, Young-Hye Cho, Young-Jin Tak, Yun-Jin Kim

**Affiliations:** Department of Family Medicine, Pusan National University School of Medicine, Busan, Korea; Biomedical Research Institute, Pusan National University Hospital, Busan, Korea; Medical Education Unit, Pusan National University School of Medicine, Yangsan, Korea; Department of Internal Medicine, Pusan National University School of Medicine, Busan, Korea; Division of Cardiology, Pusan National University Hospital, Busan, Korea

**Keywords:** Blood pressure, Hypertension, Sleep duration

## Abstract

**Introduction:**

Hypertension is a significant risk factor for cardiovascular disease (CVD). The majority of patients, however, cannot easily maintain a healthy blood pressure. Therefore, lifestyle modifications are important and may include getting enough sleep. The purpose of this study was to determine the relationship between sleep duration and hypertension, as defined by the Joint National Committee (JNC) 7 and JNC 8 guidelines.

**Methods:**

We used the data from 6,365 individuals aged ≥ 18 years based on national data from a representative sample of the 5^th^ Korea National Health and Nutrition Examination Survey V-3 in 2012. The participants were divided into three categories: JNC 7, JNC 8, and newly excluded only. The duration of sleep was classified as less than 5, 6, 7, 8, or more than 9 hours.

**Results:**

Compared with the appropriate sleep duration of 7 hours, with a sleep duration of less than 5 hours, the recommended pharmacological treatment of hypertension rate increased 1.908-fold (95% CI = 1.483-2.456) according to the JNC 8 guidelines and 1.864-fold (95% CI = 1.446-2.403) according to the JNC 7 guidelines. However, there was no statistical difference with the other sleep categories.

**Discussion:**

The recommended hypertension treatment rate increased significantly in the less than 5 hours sleep group according to the JNC 8 guidelines. To manage hypertension effectively, it may be useful to maintain a lifestyle of sleeping more than 6 hours.

## Introduction

Hypertension is a well-known risk factor of the cardiovascular system [1] that increases the risk of cardiovascular disease and places serious burdens on society and the economy [2]. Koreans have a lower risk of cardiovascular disease than that of Caucasians [3], but according to the research of Park et al. [4], the cerebrovascular disease risk for Koreans is high, and the research of Kim et al. [5] shows a high prevalence of kidney disease in the Korean population.

The most effective way to prevent hypertension-related mortality or morbidity is preventing and treating hypertension [6]. Although hypertension treatment methods have improved over the last few decades, there are still many patients that fail to reach their treatment goals. The research of Kim et al. [7] shows that Koreans are no exception. Treating hypertension is difficult and complex; therefore, to reduce the risks of developing hypertension-related disease, it is important to make lifestyle changes such as adopting a low-salt diet, maintaining appropriate weight, drinking in moderation, exercising regularly, not smoking, and dietary therapy [8,9]. In addition, the patient’s lifestyle should be reviewed for necessary modifications.

In terms of lifestyle, recent epidemiological studies suggest a minimum sleep duration to be obtained to maintain health. Research argues that short sleep duration is related to the prevalence of hypertension [6,9]_._ Sleep deprivation significantly increases blood pressure in both control and hypertension groups [10,11]. Similarly, research suggests that an appropriate sleep duration can help to lower the prevalences of hypertension, cardiovascular-related mortality, obesity, and metabolic syndrome [12,13]_._ If sleep duration is too short or too long, cardiovascular disease-related mortality increases [14]_._ Obtaining the proper amount of sleep may help prevent or treat hypertension. Kim et al. [15] argued that the prevalence of insomnia in Korea is similar to that in the western region of the continent (16 – 20%), and sleep duration in Korea tends to be shorter than that in other countries [16]_._

Past studies have analyzed hypertension patients in accordance with the former Joint National Committee (JNC) 7 guidelines. Unlike JNC 7, the new treatment guidelines (JNC 8) released in 2014 recommend that the pharmacological treatment of hypertension maintains a similar blood pressure in individuals over 60 years of age, patients with diabetes, and patients with chronic kidney disease [17]. This change in the guidelines decreases the therapeutic target blood pressure range and may influence the correlation between the recommended hypertension treatment rate and sleep duration.

The aim of the current study is to understand which populations need to consider sleep duration for the prevention and treatment of hypertension, given the known relationship between sleep duration and hypertension and the JNC 8 hypertension treatment guidelines.

## Methods

### Research subjects

This research analyzed data collected from the third survey administered by the 5^th^ Korea National Health and Nutrition Examination Survey (KNHANES V-3) in 2012 [17]. KNHANES is an ongoing national study of the health behaviors, health conditions, and food and nutritional intake of the population. For the data sampling frame of the KNHANES V (2010-2012), the 2009 population and the market price survey of apartments in 2008 was analyzed. The subjects were selected based on multistage, stratified sampling. The survey included a health interview, nutrition survey, and physical examination. The health interviews and examination surveys were conducted by mobile checkup centers, and the nutrition survey was conducted by visiting the subject’s family in person. Of the 12,722 people who were the subject of the KNHANES V-3 study, 8,057 individuals participated in the survey, which is a participation rate of 80%. KNHANES analysis were approved by Korean Centers for Disease Control and Prevention IRB and the reference number was 2012-01EXP-01-2C.

### Blood pressure measurement

Four nurses of the survey team of the Korea Centers for Disease Control and Prevention were in charge of measuring blood pressure. The nurses measured blood pressure three times manually as part of the health screening in the mobile checkup center. After the fourth survey, a review of the quality controls for blood pressure measurements found that the height of a subject’s arm might cause errors. To compensate, the measured blood pressure was adjusted accordingly. This work used the adjusted blood pressures for analysis.

### Treatment group for hypertension

#### Group following the new treatment guidelines

Using the evidence-based guidelines for hypertension treatment in adults suggested by JNC 8, [18] the pharmacological treatment recommended group was defined as follows:

1. Patients over 60 years of age with a systolic blood pressure greater than or equal 150 mmHg or a diastolic blood pressure greater than or equal 90 mmHg
2. Patients 60 years of age or younger with a systolic blood pressure greater than or equal 140 mmHg or a diastolic blood pressure greater than or equal 90 mmHg
3. Diabetes patients with a systolic blood pressure greater than or equal 140 mmHg or a diastolic blood pressure greater than or equal 90 mmHg
4. Chronic kidney disease patients with a systolic blood pressure greater than or equal 140 mmHg or a diastolic blood pressure greater than or equal 90 mmHg
5. Patients who already take hypertension medicine

#### Group defined by the existing guidelines

The treatment group that followed the JNC 7 [19] guidelines was identified as the JNC 7 group. The subjects were defined as follows

1. Patients with a systolic blood pressure greater than or equal to 140 mmHg or a diastolic blood pressure greater than or equal to 90 mmHg.
2. Patients with diabetes or chronic kidney disease with a systolic blood pressure greater than or equal to 130 mmHg or a diastolic blood pressure greater than or equal to 80 mmHg
3. Patients who already takes hypertension medicine.

#### Newly excluded only group

The subject group classified as a treatment group by the JNC 7 guidelines but excluded from the treatment group when following the JNC 8 guidelines.

### Sleep duration

The survey included in the KNHANES was used for sleep duration data. The analysis used the answers to the question, “How many hours do you sleep a day?” The duration of sleep was categorized as less than 5 hours, 6, 7, 8, or more than 9 hours.

### Statistical analysis

All data are presented as means ± SE or as prevalences (%). Sampling weights were used to account for complex sampling. For the fundamental characteristics of the groups, basic features of the treatment groups, and elemental characteristics according to sleep duration, we conducted a Pearson’s chi-square test using complex sampling in terms of the following criteria: gender, prevalence of cardiovascular disease, diabetes, chronic kidney disease, obesity, smoking, and moderate physical activity. The continuous variables for age, systolic/diastolic blood pressure, total cholesterol, triglycerides, fasting plasma glucose, and sleep duration were analyzed by linear regression analysis via complex sampling. For the two treatment guidelines that have different standards for classifying sleep duration, the odds ratio of the treatment groups was determined by logistic regression analysis via complex sampling. Statistical analysis was performed using SPSS 18.0 for Windows (SPSS, Chicago, IL, USA), and a p value less than 0.05 was considered statistically significant.

## Results

### Baseline characteristics according to treatment group

There were 6,365 respondents of the KNHANES V-3 who were over 18 years of age, and their baseline characteristics are presented in Table 1. The patients defined by JNC 7 or JNC 8 treatment guidelines were included in the treatment group, and the subjects excluded from both criteria were defined as the non-treatment group. The adults who were already taking blood pressure medication were included in the treatment group.

**Table 1:** **Baseline characteristics of all study subjects and a comparison between the treatment and non-treatment groups**

**Variables**	**Total subjects (n = 6,365)**	**Treatment group** * **(n = 1,754)**	**Non-Treatment group (n = 4,723)**	**P value**
Male sex (%)	2,099 (49.2)	767 (53.2)	1,999 (47.6)	0.051
Mean age (yr)	44.9 ± 0.3	57.5 ± 0.5	40.4 ± 0.3	<0.001
Obesity (%)	1,663 (31.7)	773 (47.5)	864 (21.1)	<0.001
Past history (%)				
CVD^†^	269 (3.4)	181 (8.4)	84 (1.6)	<0.001
DM	649 (9.7)	477 (26.1)	136 (3.0)	<0.001
CKD	40 (0.6)	16 (1.2)	24 (0.4)	0.006
SBP (mmHg)	117.0 ± 0.3	134.4 ± 0.6	111.0 ± 0.3	<0.001
DBP (mmHg)	75.5 ± 0.2	83.5 ± 0.5	72.8 ± 0.2	<0.001
Total cholesterol (mg/dL)	188.2 ± 0.7	193.0 ± 1.2	186.7 ± 0.9	<0.001
Triglyceride (mg/dL)	131.3 ± 2.1	170.3 ± 4.9	117.0 ± 2.0	<0.001
FPG (mg/dL)	97.1 ± 0.4	107.8 ± 1.0	93.0 ± 0.3	<0.001
Current smoking (%)	743 (20.6)	227 (18.5)	507 (21.2)	0.087
Physical activity^‡^ (%)	791 (17.6)	235 (14.1)	734 (21.7)	0.001
Sleep duration (hours)	6.8 ± 0.29	6.7 ± 0.04	6.9 ± 0.03	<0.001

Values are presented as means ± SD or frequencies (%). P-value for treatment group vs. non-treatment group.

*Treatment group included participants taking antihypertensive medication.

^†^CVD included myocardial infarction, angina, and stroke.

^‡^Physical activity, moderate exercise at least three times a week.

CVD, cardiovascular disease; SBP, systolic blood pressure; DBP, diastolic blood pressure; FPG, fasting plasma glucose; DM, diabetes mellitus; CKD, chronic kidney disease.

Compared with the non-treatment group, the treatment group showed a significantly higher ratio of men and higher age. Systolic and diastolic blood pressures as well as total cholesterol, triglycerides, fasting plasma glucose, and diabetes prevalence were all higher than those of the non-treatment group. The prevalence of chronic kidney disease and obesity were also higher. In the case of moderate exercise, the number of subjects was significantly fewer, and they had a shorter sleep duration.

### Comparison between JNC 8 and Newly excluded only in the treatment group

There were 193 people defined by the JNC 7 guidelines, but not by the new guidelines, as a treatment group; these individuals were designated as newly excluded. A comparison of this group with the new treatment guidelines group did not show significant differences in systolic blood pressure, but the JNC 8 guidelines group had a higher diastolic blood pressure. Diabetes and fasting plasma glucose levels were higher in the newly excluded only group. The JNC 8 guidelines group had a significantly greater prevalence of obesity (Table 2).

**Table 2:** **Comparison between the JNC 8 and newly excluded only treatment groups**

**Variables**	**Treatment category** *			
	**JNC 7 (n = 1,754)**	**JNC 8 (n = 1,561)**	**Newly excluded only (n = 193)**	**P value**
Male sex (%)	767 (53.2)	54.1	45.9	0.101
Mean age (yr)	57.5 ± 0.5	57.9 ± 0.6	56.2 ± 1.5	0.365
Obesity (%)	773 (47.5)	702 (48.8)	71 (36.8)	0.012
Past history (%)				
CVD^†^	181 (8.4)	174 (8.9)	7 (4.1)	0.129
DM	477 (26.1)	382 (23.1)	95 (50.5)	<0.001
CKD	16 (1.2)	21 (1.2)	3 (1.3)	0.864
SBP (mmHg)	134.4 ± 0.6	134.3 ± 0.6	135.7 ± 1.1	0.268
DBP (mmHg)	83.5 ± 0.5	83.9 ± 0.6	80.3 ± 0.7	<0.001
Total cholesterol (mg/dL)	192.9 ± 1.2	192.3 ± 1.4	198.0 ± 4.1	0.216
Triglyceride (mg/dL)	170.3 ± 4.9	166.6 ± 4.5	200.4 ± 28.6	0.249
FPG (mg/dL)	107.8 ± 1.0	105.6 ± 1.0	125.2 ± 4.1	<0.001
Current smoking (%)	227 (18.5)	201 (18.3)	26 (20.0)	0.737
Physical activity^‡^ (%)	235 (4.1)	212 (14.5)	23 (10.9)	0.249
Sleep duration (hours)	6.7 ± 0.03	6.7 ± 0.04	6.8 ± 0.20	0.427

Values are presented as means ± SD or frequencies (%). P-value for JNC 8 vs. newly excluded only.

*Treatment category; JNC 7 included participants taking antihypertensive medication and content with JNC 7 guidelines; JNC 8 included participants taking antihypertensive medication and content with JNC 8 guidelines; Newly excluded only included participants treated under JNC 7, but not JNC 8, guidelines.

^†^CVD included myocardial infarction, angina, and stroke.

^‡^Physical activity, moderate exercise at least three times a week.

CVD, cardiovascular disease; SBP, systolic blood pressure; DBP, diastolic blood pressure; FPG, fasting plasma glucose; DM, diabetes mellitus; CKD, chronic kidney disease, JNC, Joint National Committee.

### The baseline and metabolic characteristics of all study subjects classified by sleep duration

Table 3 shows the basic features of the population groups categorized by sleep duration. The largest group had 1,372 individuals with a sleep duration of 7 hours. There were 793 individuals in the group with a sleep duration of 5 hours or less. There were 362 individuals in the group with a sleep duration of 9 hours or more. The group with 5 hours or less sleep showed the highest rate of cardiovascular disease and were of the highest average age. The systolic and diastolic blood pressures were also higher in this group than any other group, and the fasting plasma glucose level was significantly higher. The 7-hour sleep duration group had the highest ratio of males.

**Table 3:** **Baseline and metabolic characteristics of all study subjects classified by sleep duration**

**Variables**	**Sleep duration (hours)**					
	**≤5 (n = 793)**	**6 (n = 1,284)**	**7 (n = 1,372)**	**8 (n = 1,164)**	**≥9 (n = 362)**	**P value**
Male sex (%)	240 (39.1)	535 (52.8)	567 (52.3)	470 (49.1)	137 (41.8)	<0.001
Mean age (yr)	53.0 ± 0.9	44.5 ± 0.6	44.0 ± 0.5	42.9 ± 0.6	43.3 ± 1.3	<0.001
CVD* (%)	73 (6.2)	60 (3.2)	51 (2.5)	63 (3.4)	17 (3.0)	0.004
SBP (mmHg)	121.3 ± 0.9	117.8 ± 0.6	116.8 ± 0.6	116.5 ± 0.6	116.0 ± 1.0	<0.001
DBP (mmHg)	76.3 ± 0.6	76.3 ± 0.5	76.1 ± 0.4	75.3 ± 0.5	72.9 ± 0.9	0.005
Total cholesterol (mg/dL)	190.2 ± 1.7	187.9 ± 1.4	190.2 ± 1.4	186.1 ± 1.3	187.3 ± 3. 3	0.126
Triglyceride (mg/dL)	133.3 ± 5.1	129.5 ± 3.3	135.0 ± 4.4	129.4 ± 4.8	135.0 ± 7.0	0.748
FPG (mg/dL)	99.3 ± 0.9	96.3 ± 0.7	97.4 ± 0.8	96.3 ± 0.8	95.3 ± 1.2	0.018
Obesity (%)	277 (34.2)	428 (33.3)	422 (32.5)	333 (29.5)	104 (28.9)	0.303
Current smoking (%)	80 (16.1)	202 (22.8)	233 (23.1)	174 (19.1)	52 (20.7)	0.081
Physical activity^†^ (%)	112 (16.2)	214 (17.5)	239 (20.4)	168 (15.1)	51 (17.3)	0.120

Values are presented as means ± SD or frequencies (%). P-value for sleep duration category.

*CVD included myocardial infarction, angina, and stroke.

^†^Physical activity, moderate exercise at least three times a week.

CVD, cardiovascular disease; SBP, systolic blood pressure; DBP, diastolic blood pressure; FPG, fasting plasma glucose; DM, diabetes mellitus; CKD, chronic kidney disease.

### The odds ratios for the rate of recommended hypertension treatment according to the sleep duration category

Compared with the group with 7 hours of sleep, the recommended treatment rate of hypertension was significantly higher with a sleep duration of 5 hours or less according to the JNC 7 guidelines (OR = 1.864, 95% CI = 1.446-2.403). However, the rest of the JNC 7 categories did not show any significant difference in hypertension rates (Table 4). This trend was also seen with the JNC 8 guidelines for a sleep duration of 5 hours or less (OR = 1.908, 95% CI = 1.483-2.456).

**Table 4:** **Odds ratios for the prevalence of hypertension according to sleep duration category**

**Prevalence** *	**Sleep duration (hours)**				
	**≤5**	**6**	**7**	**8**	**≥9**
JNC 7	1.864(1.446-2.403)	0.973(0.785-1.206)	1.00 (reference)	0.947(0.752-1.193)	0.954(0.692-1.315)
JNC 8	1.908(1.483-2.456)	1.009(0.811-1.255)	1.00 (reference)	0.971(0.776-1.215)	0.988(0.708-1.379)

*Prevalence, prevalence of hypertension; JNC 7 included prevalence of hypertension according to JNC 7 guidelines and the subjects taking antihypertensive medication; JNC 8 included prevalence of hypertension according to JNC 8 guidelines and the subjects taking antihypertensive medication.

JNC, Joint National Committee.

## Discussion

Hypertension is one of the major causes of death. The prevention and treatment of hypertension are key to decreasing overall mortality and morbidity rates [20,21]. However, the number of hypertensive patients who maintain their blood pressure under the target is not that high [6]. For this reason, lifestyle modifications should be practiced. One new lifestyle modification is obtaining enough sleep [5,9-11]. Other researchers have reported that too short a sleep duration increases blood pressure and the prevalence of hypertension. JNC 7 proposed treatment guidelines for hypertension in 2004. JNC 8 was established in 2014 [17,19], the new treatment guidelines of which are based on newly reported, randomized control experiments, and these guidelines permit a higher systolic blood pressure.

Past research on the relationship between sleep duration and hypertension was based on the JNC 7 guidelines. The question of whether the relationship between hypertension and sleep duration is still maintained under the JNC 8 guidelines prompted this research. In this study, we examined the correlation between sleep duration and the recommended treatment rate of hypertension using the JNC 8 criteria. An earlier study reported a hazard ratio of 1.20 (95% CI = 1.09-1.31) for short sleep duration on hypertension prevalence, and this study suggests 7 hours of sleep to be the appropriate duration [22]. One meta-analysis reported that the odds ratio for hypertension risk due to a shorter sleep duration is 1.20 (95% CI = 1.09-1.32). Based on other reports, too much sleep can also increase hypertension risk (OR = 1.11, 95% CI = 1.05–1.17) [23]. Another study found that short sleep duration increased the prevalence of hypertension, but long sleep duration had no statistically significant difference [24]_._

In the current study, we defined the standard duration of sleep as 7 hours in accordance with recent research [25,26] A sleep duration of less than 5 hours increases the need for hypertension treatment by 1.864-fold (95% CI = 1.446-2.403) according to the JNC 7 guidelines, and by 1.908-fold (95% CI = 1.483-2.456) according to the JNC 8 guidelines. Both guidelines by JNC 7 and JNC 8 showed that short sleep duration increases the rate of recommend treatment of hypertension alike, which are very similar to the former results [22,23]. JNC 8 tends to exclude obesity or diabetes cases in their estimates, which were considered under the JNC 7 guidelines. Because of these excluded groups, the prevalence of cardiovascular disease and diastolic blood pressure was higher with the former guidelines. Despite the differences among the subjects, a sleep duration of less than 5 hours increased the hypertension risk by similar levels. However, a long sleep duration did not correlate with the prevalence of hypertension. Compared with other countries, this difference may be related to the shorter sleep duration of Korean individuals. Together, these results support the recommendation for a sleep duration of 6 hours or more among Korean hypertension patients.

The activation of the sympathetic nervous system explains the mechanism linking short sleep duration to hypertension prevalence. As sleep duration shortens, the waking state is maintained and the sympathetic nervous system stimulated. This also increases nighttime blood pressure levels. An increased heart rate and elevated salt intake also present a risk of hypertension [6,27].

This study’s findings may be generalized to a larger population, because it analyzed representative data among a diverse and large group of Koreans. It also determined the appropriate amount of sleep in accordance with the treatment guidelines suggested in 2014. However, there are some limitations to how well the study findings can be applied to the general population. First, this research is based on a cross-sectional survey; thus, while it may suggest a correlation between hypertension and sleep duration, it cannot identify the causal link with any certainty. Second, the survey on sleep duration was based on one question. In addition, there was a report suggesting that the quality of sleep influences the hypertension prevalence more than does the quantity of sleep [6,28,29], and this was not addressed in the current study. Third, even though the importance of this association was weakened by the JNC 8 guidelines, 24-hour blood monitoring data are still significant for assessing blood pressure status. However, the KNHANES V3 data did not include 24-hour blood monitoring data, which limits our study. Finally, the proportions of males and older patients were higher among participants with short sleep duration. Gender and age were two of the most significant factors influencing hypertension; the impact of sleep duration may be minor for the treatment and management of hypertension. However, gender and age are not modifiable, and although the lifestyle modification of increased sleep may have only a weak effect, it still warrants recommendation due to even this small influence.

## Conclusions

In conclusion, there was a statistically significant increase in the rate of recommended hypertension treatment among individuals with a short sleep duration, regardless of whether the hypertension treatment guidelines were according to JNC 7 or JNC 8. To control hypertension, it is useful to modify lifestyle to maintain a proper sleep duration of more than 6 hours. This study is limited by data collected via a cross-sectional survey that did not investigate sleep sufficiently. A prospective study is needed to overcome these limitations.
